# Doing ‘reciprocity work’: The role of fieldworkers in a mass drug administration trial in the Gambia

**DOI:** 10.1080/17441692.2022.2125998

**Published:** 2022-10-02

**Authors:** Alexandra Fehr, Claudia Nieto-Sanchez, Joan Muela, Ebrima Manneh, Dullo Baldeh, Omar Ceesay, Umberto D’Alessandro, Edgard Dabira, Patricia Kingori, Koen Peeters Grietens, Azucena Bardají, Joske Bunders-Aelen, Teun Zuiderent-Jerak

**Affiliations:** a Athena Institute, Vrije Universiteit Amsterdam, Amsterdam, Netherlands; b Unit of Socio-Ecological Health Research, Institute of Tropical Medicine Antwerp, Antwerp, Belgium; c Medical Anthropology Research Centre, Universitat Rovira I Virgili, Tarragona, Spain; d Medical Research Council Unit The Gambia, Banjul, Gambia; e The Ethox Cetre, University of Oxford, Oxford, UK; f IS Global, Barcelona Institute for Global Health, Barcelona, Spain

**Keywords:** Fieldworkers, mass drug administration, malaria, research ethics, the Gambia

## Abstract

In their roles as nurses, data collectors, or other, fieldworkers undertake myriad tasks working intimately with and on the bodies of others – a type of work called ‘body work’. This work further includes the micropolitical relations shaping these interactions, and studies have shown the importance of these relationships in the success of clinical trials, particularly in the Gambia. This study seeks to expand the concept of body work to understand the roles and interactions of fieldworkers within the trial community, and the effect on a mass drug administration (MDA) clinical trial. We conducted a mixed-methods social science study alongside the MDA in 2018–2019, including indepth interviews, focus group discussions, and semi-structured observations with the population involved (and not) in the MDA, as well as the MRC fieldworkers. We found that fieldworkers participated in what we call ‘reciprocity work’. Through their regular tasks and interactions, they necessarily showed respect and established trust in a way that formed and contributed to reciprocal relationships, the results of which impacted the trial and individuals’ autonomy in the decisionmaking process. Understanding the role of fieldworkers and their reciprocity work is a vital component in comprehending how research ethics are made and conducted in global health research.

## Introduction

The recent rise in literature regarding the decolonisation of global health research and practice has helped to highlight the strong and historic power and colonial relationships that exist in the discipline, particularly between large health institutions (often based in the Global North) and the communities in which they work (most often based in the Global South) (Chaudhuri et al., 2021). Much continues to be written about how global health organisations and institutions can change the current power paradigm, and many of the recommendations include greater involvement of target communities in the design and decision-making of health interventions or clinical trials (Khan et al., 2021; Vincent et al., 2022). Regardless of which community engagement strategies are used to carry this out, greater involvement of the local communities will require the work and skills of the frontline and fieldworkers who are often saddled with the task of mediating between research institutions and communities as is (Kingori, 2013; Vincent et al., 2022).

Fieldworkers can include data collectors, field nurses and others who work on the ‘frontline’ of doing and generating Global Health research and knowledge. Due in part to their positions ‘in the field’, fieldworkers remain an underrepresented cadre of the Global Health workforce, out of view from the gaze of the discipline’s leadership and policy makers (Kamuya et al., 2013; Kingori, 2013; Maes, 2015; Molyneux et al., 2010). During clinical trials, the specific work required of fieldworkers causes them to be intimately involved in and with the lives and bodies of potential trial participants, a type of work referred to as ‘body work’ (Twigg et al., 2011). Body work is a form of paid labour undertaken by a vast variety of healthcare and social workers focused on the ‘assessing, diagnosing, handling, treating, manipulating, and monitoring’ of bodies (Twigg et al., 2011). In this sense, body work is the type of labour that most fieldworkers are trained and hired to do in the context of clinical trials; they take necessary body measurements, administer doses of medication, and respond to and record any reactions of trial participants.

However, beyond the corporal, body work also includes the emotions, time and space required of this work, as well as the ‘micro-political relations’ between those doing the work and those being worked on (Twigg et al., 2011). Just as essential to their positions as the physical components, fieldworkers must also work within the micro-political relations of body work. They do this, in part, by maintaining regular face-to-face interactions with those from the trial communities and working within the context of their own relationships with the individuals in them (Gikonyo et al., 2008; Kamuya et al., 2013). This requires fieldworkers to have unique skillsets, including tacit skills and ‘local knowledge’. This is not only desired by research institutions (Kingori & Gerrets, 2019) but also requires fieldworkers to navigate the everyday ethics of clinical trials, managing – and potentially compromising – their own ethical beliefs (Kamuya et al., 2015; Kingori, 2013). Studies have demonstrated that these additional skills – which may include participating in relationships of reciprocity, establishing trust and showing respect – are not skills included in job descriptions or trainings, but are nonetheless essential to the success of clinical trials (Dada et al., 2019; Geissler et al., 2008).

Within the Gambia, and the Medical Research Council Unit the Gambia (MRCG) in particular, the role of the fieldworkers is paramount (Fehr, Muela, et al., 2021; Fehr, Nieto-Sanchez, et al., 2021; Kelly, 2011; Leach & Fairhead, 2011). The MRCG, a faculty of the London School of Hygiene and Tropical Medicine, was founded in the Gambia in 1947–1918 years before the country’s independence – and is the United Kingdom’s largest international research institution (Medical Research Council Unit the Gambia, 2018). Working with academic and government partners, MRCG’s mission is to improve the health of West Africa by focusing on disease control and elimination, nutrition, vaccines and immunity and maternal and child health, as well as to provide clinical and scientific training (Medical Research Council Unit the Gambia, 2018).

Social science studies, often conducted concurrent to the institution’s clinical trials, have demonstrated the unique and complex history and relationship MRCG has in the Gambia and with the villages where studies and interventions are conducted (Fehr, Muela, et al., 2021; Kelly, 2011; Leach & Fairhead, 2011). Within its history, MRCG has been present through times of turmoil and insufficient government resources, including the political and economic crises of the 1990s. During this time, there was a decline in government-provided healthcare and an increase in foreign scientific research – particularly through the MRCG. By way of its clinics and the ancillary care provided during clinical trials, the MRCG has been the *de facto* healthcare option most readily available to many Gambians, especially those in rural areas (Geissler et al., 2008; Leach & Fairhead, 2011). Therefore, most of those enrolled in MRCG trials regard the institution as one of healthcare provision or foreign aid rather than research (Geissler et al., 2008; Leach & Fairhead, 2011). For those living in trial villages, clinical trials are not necessarily viewed as specific and time-bound activities; rather, people see the work of MRCG as a continuous effort to ‘work with’ the village and provide necessary medical care that is otherwise inaccessible (Geissler et al., 2008; Kelly, 2011; Leach & Fairhead, 2011).

This historical setting of power and resource inequities has created complex relationships between the MRCG and the trial villages, but how this relates to and impacts the fieldworkers themselves is under explored. As part of their role, MRCG fieldworkers are engaged in complex relationships with the individuals in trial communities. This includes relationships of ‘exchange’ (Geissler et al., 2008), that have been shown to directly impact trial enrolment, informed consent and continued trial participation (Kelly, 2011; Kelly et al., 2010). The relationships between institutions and communities are bound to change as Global Health works to decolonise its research and practice. As these relationships are mediated through the fieldworkers, it is ever more important for research institutions and those who make Global Health ethics and policy to understand the complex roles of the fieldworkers, and how their body work may be affected, as they navigate these changing dynamics. Working within this context, the objective of this study is to expand the notion of body work, particularly the micro-political relationships it includes, to understand the roles, interactions and effects of the fieldworkers on the individual participants and the overall success of a mass drug administration (MDA) trial for malaria elimination in the Gambia.

## Methods

### Study setting

This study took place in the southern bank of the Upper River Region (URR) of the Gambia, an area with seasonal malaria transmission patterns, and the highest rates of malaria in the country. In this setting, each village is headed by the Alkalo, the village chief, and is further divided into compounds, each led by the compound head (usually the oldest male of the family). Subsistence farming is the most prevalent economic activity in the area, though some villagers also engage in herding, fishing, or small businesses. Importantly, government-provided healthcare is difficult to access, and private healthcare is virtually unattainable. Trial villages are located far from government health facilities, and transportation is a prohibitive factor in terms of access. Further, health facilities are often subject to stock-outs, forcing people to try and obtain expensive treatments from private pharmacies.

### Study design

We conducted a mixed-methods ethnographic social science study concurrent to the ‘Mass drug administration of ivermectin and dihydroartemisinin-piperaquine as an additional intervention for malaria elimination (MASSIV)’ trial, a community-based, cluster-randomised control trial that took place in 2018–2019 (Dabira et al., 2020). The objective of the overall social science study was to understand social factors influencing the effectiveness of the MDA intervention, including acceptability and its effects on coverage and adherence. This paper focuses on the roles of the fieldworkers and their interactions in the trial villages.

The MASSIV trial was conducted by the MRCG and international partners, including the London School of Hygiene and Tropical Medicine and the Institute of Tropical Medicine Antwerp. The MDA trial took place in 16 control and 16 intervention villages. The overall target population for the MDA was approximately 5400 people; each trial village ranged in size from 140 to 700 people. In the intervention villages, the trial consisted of directly observed therapy (DOT) of the two MDA trial medications. Medication was to be taken three days in a row for three consecutive months prior to the peak of malaria transmission season (August–October in 2018 and July–September in 2019). In the control villages, those who opted to enrol were asked to provide a blood sample for malaria testing. No trial medications were distributed in the control arm except in the event of malaria-positive cases. Outside the sensitisation meeting, the MASSIV trial had no formal community engagement activities.

### Data collection

This study included in-depth interviews (IDIs) and focus group discussions (FGDs), as well as informal conversations with who did and did not take the MDA medications, village leaders and stakeholders, and MASSIV field staff in both the control and intervention arms of the trial. Sampling was purposive to ensure maximum variation in level of involvement in the trial. Structured and semi-structured observations of all components of the MDA trial also took place, from pre-trial sensitisation meetings through the final drug distribution.

For this specific study, additional IDIs were conducted with fieldworkers throughout the duration of MASSIV. Though the population of interest included nurses of different levels and specialities, data collectors and others, we have chosen to use the general term ‘fieldworker’ throughout the manuscript to protect the anonymity of respondents. Interviews took place where convenient and private, such as in the evening after drug distribution in the trial villages, or in between distribution rounds. In addition, the social science team spent multiple days and nights living with trial fieldworkers in the intervention villages during the MDA and conducted multiple additional structured observations and informal conversations. At the end of each day with the fieldworkers, the social science team in the field held in-depth discussions on their observations and informal conversations. Detailed notes were taken during these discussions and typed up to be included in the analysis process.

### Data analysis

The analysis of data was a continuous, flexible, and iterative process, and regular reflexive discussions were held with the social science team in the field and abroad. These methods ensured a thorough understanding of emerging issues and concepts; they lasted throughout data collection and analysis until data saturation was reached and no new themes emerged. IDIs and FGDs with those in the trial villages were conducted in the local language (either Fula or Mandinka) and were recorded, transcribed verbatim and translated into English with the aid of trained field staff. IDIs and informal conversations with the fieldworkers were conducted in English; IDIs were recorded and transcribed verbatim. Qualitative data were analysed using NVIVO v12 software (NVivo, 2018).

### Ethics

Ethical approval for this study was granted by the Institutional Review Board of the Institute of Tropical Medicine in Antwerp, Belgium, and by the Scientific Coordinating Committee and Ethics Review Board of the Medical Research Council Unit the Gambia. All respondents provided verbal informed consent prior to participation.

## Results

Results are based on over 200 IDIs and 29 FGDs, as well as many informal conversations and nonrecorded interviews. These included 12 formal IDIs, 1 FGD, and myriad informal conversations and observations specifically with and of the MASSIV field staff.

### The MASSIV fieldworkers

With the exception of one fieldworker in Year 1 of the MDA, all MASSIV fieldworkers were men. They ranged in age from early-20s to mid-50s and originated from all regions of the Gambia (though very few were from the specific trial area, and none originated from the trial villages). Most, but not all, fieldworkers were trained as nurses, and all of them had previously worked with MRCG in similar roles on other clinical trials; a few had worked with MRCG for several decades. Importantly, due to their long-term employment with MRCG, some of the fieldworkers had experience in several of the MDA trial villages and were already familiar with the families and individuals who lived there.

Throughout the MDA trial, fieldworkers had many roles, responsibilities and tasks that expanded well beyond the summary of only their physical body work that is depicted here. At the beginning of the trial, one of their first tasks was to visit the intervention and control villages and speak to the Alkalos about the trial and what they could expect for their village. The intent of this meeting was also to get the Alkalos’ permission for the trial to take place in their village; no Alkalo turned down participating in the MDA trial. At these initial meetings, the Alkalos and fieldworkers would also confirm a time for the fieldworkers to return to the villages and conduct the sensitisation meetings. During sensitisation meetings, fieldworkers used the participant information sheet as a speaking guide to ensure they told the communities consistent information about all aspects of the MDA trial. At the end, they would answer any questions posed by the community members or leaders. A few days after the sensitisation meetings, fieldworkers returned to the villages in teams (the size of which was dependent on the village population) and, travelling compound-to-compound, conducted the informed consent and enrollment processes. This included providing detailed information on the MDA trial to the individuals of the compound; the audience was often comprised of the compound head, his sons and his first wife. Once they gave their initial consent, the fieldworkers obtained informed consent and enrolled the rest of the compound members who were willing to take part in the MDA trial.

On the days of DOT in the intervention villages, fieldworkers continued to engage in much of the physical aspects of their body work. They would arrive, set up their distribution centre, distribute medications and record all the trial data into a tablet. At the beginning of the trial, this included height and weight for dose calculations, as well as medical histories to determine eligibility. At the start of each round, this also included pregnancy tests for women and girls of reproductive age. If necessary, some fieldworkers would try and encourage those in the villages to come take the medication or would move compound-to-compound with the medication in an attempt to catch those who had not yet taken it. Importantly, in Year 2 of the MDA, fieldworkers were instructed by trial leadership to be much more present in the trial villages. Therefore, fieldworkers lived, slept and ate with hosts throughout the DOT – engaging in the physical and non-physical aspects of body work – and one or two would stay in the village the following week to be present in the event of any adverse events.

### Fieldworkers and the MRCG-community relationship

On the surface, the long-lasting relationship between the MRCG and the trial villages was readily apparent. Though respondents were aware of the details of the specific MDA that was taking place, most did not differentiate it as a separate trial and, in fact, viewed it as MRCG continuing to ‘*work with*’ their village. Important to respondents in a context where access to medical care is limited or non-accessible, many in the villages expressed that MRCG has long been their primary source of healthcare. Respondents provided numerous examples of how MRCG had treated a sick child or provided transport to the health facility for pregnant women in labour – all without charge to the individual.

Respondents nearly always referred to the institution as being the one to provide resources and work with the village; there was no particular role of individual fieldworkers. And though there were many people more ‘senior’ than them in the MDA trial leadership and implementation of the trial, it was evident in their day-to-day interactions that the individual fieldworkers in the villages represented the whole of the MRCG for the villagers. We will describe several instances reflecting this perception.

First, due to its history of providing care, many respondents expressed a need to reciprocate to the MRCG by participating in the MASSIV trial. For example, one man in a control village recounted an incident from several years’ ago when his son was suffering from severe malaria. After not being able to access care or medication at the local health facility, the MRCG successfully treated the boy, free of charge. When asked of his motivations for providing a blood sample as part of the control arm of the trial (where he would not receive the MDA medication), the man responded, *‘because MRC[G] saved my son, I will do whatever they ask of me’*. In this case, the fieldworker asking for the blood sample was a representation of the MRCG, and by complying with his request to provide a blood sample, the man felt he could give back to the institution for having cared for his son.

In addition, respondents thought about the effects of their present interactions with the MRCG on their future, as well as the future of the villages’. In interviews throughout the study, it was common for respondents to answer our final questions – *‘is there anything else you would like to add?’* and ‘*do you have any questions for us?*’ – by saying how grateful they were for the work of MRCG and that they hoped MRCG would continue *‘working with’* their village. Many further specified that they wanted MRCG to notice the high levels of participation in the MDA trial with the expectation that the institution would later reciprocate with additional projects. For example:

Interviewer: ‘Besides the benefit of the medicine, are there any other reasons why [Village] had many people come and take the medicine?’

Respondent: ‘We like the MRC[G] and we would like our village to develop. We trust the MRC[G] … It is good to take the medicine because it is in our own interest so that MRC[G] would continue operating in this area. – Focus group discussion with adolescent-aged girls.

Second, the historical representation of MRCG was also true in the event of negative behaviour or trial-related problems. In the first year of the MDA, communication issues meant it was not guaranteed that the fieldworkers would be provided with extra medications, such as paracetamol, to alleviate potential side effects of the trial medication (e.g. headache) (this issue was later addressed and remedied in subsequent rounds). The fieldworkers recognised this as problematic for several reasons. First, not providing these medications could erode the communities’ trust in them and affect their relationships with individuals during the course of this MDA trial. Second, it could also erode the communities’ trust in the MRCG, which could affect future trials. Therefore, there were multiple examples of fieldworkers purchasing these medications with their own money.

‘When you are doing research, there are certain drugs available for you to counter any reaction or anything that your people complain about - at least you should give them those - because you are using them … paracetamol or anything like that sort thing that they can use to relieve whatever pain they encounter. As far as whenever you are doing a trial on a drug, whatever you issue to them, whatever condition they have, it is really associated with the [trial] drug … [Having paracetamol, etc.] encourages them to continue taking the drug and it will also have them accept any other drug that is going to be tried in their village here, because when we do good to them and make sure we’re taking care of them, they are happy - whatever drug is being brought in for trial in to their community, they will really accept, because they will think about the past situation that they encountered. If that was good, they will take it up, but when it is bad, you will start seeing some refusals. – Fieldworker.

Third, just as fieldworkers represented MRCG, the Alkalos represented their villages in the MRCG-Village reciprocal relationship. In many interviews with our team, Alkalos emphasised the ongoing relationship their village has with MRCG and that MASSIV was another project with which they were happy to be involved. This message was echoed at the pre-trial community sensitisation meetings, where they would speak on behalf of the village to the fieldworker(s) communicating on behalf of MRCG. During these events, the Alkalos would emphasise the villages’ general support and appreciation for MRCG, as well as their expectations of high MDA coverage from the community. Like those in their village, the Alkalos directed expressions of hope to the fieldworkers that this would lead to continued interventions, particularly those focusing on other development projects (periodically including those outside the functions of MRCG, such as the provision of water and electricity).

### Fieldworkers and individual relationships

Fieldworkers spent a lot of time and occasionally resided in trial communities during the MDA (especially during DOT). As such, they developed individual relationships with people in the villages and it was important they interacted in socially appropriate ways. While carrying out the full responsibilities of their corporal body work – predominantly administering and recording MDA medication – they also balanced these social expectations and micro-political relations. Two of the most important expectations for peacefully living in the villages and achieving high MDA coverage were showing respect and establishing trust.

### Showing respect

Showing respect was vital to the success of the MDA trial and began even before the first sensitisation meeting took place. As representatives of MRCG, it was essential for fieldworkers to show respect to the Alkalo, as the village leader, in their very first meeting. Often this meant bringing a small offering of kola nuts, a traditional gesture of respect, and greeting him properly. If respect was not shown at these initial meetings, and the Alkalo was not happy with the initiation of the relationship, the trial would never begin in the first place, and the Alkalo may ‘*chase you out of the village’*. For each subsequent visit to the village, it was then expected that the fieldworkers would first greet the Alkalo before any other work took place.

After the Alkalo, appropriately and respectfully entering individual compounds was another essential aspect of showing respect; this process included recognising the leadership role of the compound head. Multiple compound heads told us during Year 2 of the trial that they noticed and highly appreciated the increase in respect they were shown by the fieldworkers and that this increased their likelihood of participating in the trial. A prime example of this occurred during the informed consent and enrollment process (which was conducted again before the start of Year 2). When a fieldworker entered a compound and found that the compound head was not at home, rather than consenting and enrolling those adults and children who were present – as was stated in the trial protocol – they would skip the compound all together and only return when the compound head was present. This allowed the compound head to give his consent for the compound as a whole before anyone else gave their consent. As one compound head reported at the beginning of the second year:

The consenting this year is very good as I was made to understand that all the project is about malaria, and this time the fieldworker or the nurse who talked to me spoke good Fula. And this time around, they waited for me to come and discussed with me rather than what they did last year – they entered my compound without my consent … This year the consenting is done the way I wanted it, as they have shown me that I am the compound head and waited for me till I came [back to the compound] and talked with me, rather than doing what they did last year - talking with my wives – which I don’t agree with. I am the compound head; I give orders to what my family should do. – Compound head, MDA Year 2.

In the event a fieldworker did not follow these social norms, even if they contradicted the trial protocol, village members would be unhappy and the relationship would potentially be disrupted; further, it would risk an individual’s participation. For example, one young woman over the age of 18 explained that a fieldworker came to her compound for the informed consent and enrollment process when her mother was not at home. The woman told the fieldworker that she could not give consent to enrol in the trial without first asking her mother. The fieldworker then told the woman that because she is an adult, she can provide consent on her own. The woman was very unhappy with the lack of respect in this response and declined participation. When her mother returned, the woman told her of the incident and they both declined any further involvement in the MDA trial.

This respect continued to be crucial throughout the MDA trial activities and each time a fieldworker interacted with a village member – especially if they were new to the trial. As one fieldworker explained to us, this initial greeting, ‘*the approach*’, required respect and acknowledging the importance of the individual’s role in the trial:

[Regarding] the respect that we show to the participants [during our] approach [in asking them to participate in the trial], you know, [is to focus on] their importance of participating in this program. When they come, we approach them in a respectful manner. We receive them and then we try to educate them more and [explain] to them their importance of participating in this program and what participation is for them and for the project, overall, for the community at large. So, their role as far as activities is concerned is of great importance and they deserve that respect to be given to them. So, we treat them as participants who are providing very valuable information for the project, who are contributing to the success of the project. – Fieldworker.

The inequalities between fieldworkers and those in the villages were widely recognised. Fieldworkers were far more likely to be educated and have steady employment, and they had greater access to material goods and resources. An important way of showing respect was for the fieldworkers to not act ‘*too big*’ and to ‘*come to their level;*’ it was important to behave in a way that demonstrated they were not somehow better than those in the villages. This could have been a strategic move by a fieldworker or a way to increase a sense of belonging. Regardless, this required work and knowledge beyond what was stated in their job description. Common ways for fieldworkers to show this type of respect included making it a point to be more actively involved in the community and the lives of the individuals in ‘their’ villages. For example, a (non-trial related) death occurred between MDA rounds in one of the villages. The fieldworkers of that village attended the funeral in their own time and made the customary monetary donations from their own salaries. The village greatly appreciated this sign of respect and the fieldworkers’ involvement with and care of the village. At other times, fieldworkers would attend naming ceremonies or other village activities, or during slow times of the MDA, fieldworkers would engage in playful activities, making jokes and entertaining those in the village. These actions, this extra body work, helped secure the fieldworkers’ positions as part of the communities and were vital to the success of the MDA trial.

### Establishing trust

*‘If there is no respect, there is no trust’*, was a highly acknowledged sentiment among the MASSIV fieldworkers. But establishing trust required much more than showing respect on behalf of the institution they represent. For some fieldworkers, this included maintaining an active, continuous dialogue with community members throughout the trial. This could include providing details of the MDA, the importance of the medication, or the greater implications of the trial results. It was also an opportunity for community members to ask questions and for fieldworkers to dispel rumours.

… Another thing, like the day before the actual MDA, I would be going to compounds telling them about the project so that they can understand, because what I have realized on the way is like most of them, they just think that this thing is for treatment not realizing that is research. They do not understand what is the difference between research and treatment. So, you need to make them to understand and the benefit of this trial … So, with that, many people started to understand. They all post their questions to me; the ones I can answer, I answer. The ones I cannot answer, I refer them to a later date and when I have answers for that, I would answer them. I would also show them the potential side effects expected because these are some of the things that makes them to withdraw, because if you do not tell them the side effects, if they got it they would think that the drug is giving them problems, but if you tell them prior to the MDA, if they see the side effect, they would expect that this was the side effect this man was telling us. – Fieldworker.

Fieldworkers would also utilise their ‘localness’ to establish trust in the trial villages. Though most were from elsewhere in the country, there was a shared idea that ‘*we are all Gambian*’. Historically, rumours have existed in the Gambia that the MRCG steals and sells blood to white foreigners. We were told these rumours have substantially declined, and they were rarely acknowledged among respondents, with the exception of their existence in the past or among small groups of *‘uneducated’* people. Being part of the same national identity, however, garnered trust in the fieldworkers and a knowingness that they would not cause harm to their fellow brothers and sisters. In addition to not stealing blood, this also implied the safety – and potential efficacy – of the MDA medications and may have encouraged some to take part in the MDA trial.

Fieldworkers are often employed through project or trial-specific contracts, and many of the MASSIV fieldworkers had worked continuously for years moving from one contract to the next. They strongly felt the need to demonstrate that they had successfully completed their work to ensure they were in good standing and, therefore, able to easily move to the next contract and continue their employment without interruption. These additional strategies and extra-job activities used to show respect and establish trust were recognised by the MASSIV fieldworkers as essential to achieving the indicators of job success, such as high enrollment and coverage rates.

I think respecting the community can make them to come out and drink the medicine which I think the MRC staff are practicing by respecting the elders, by giving them seats when they come to take the medicine, and we always greet them anywhere we meet … That has a good effect for the coverage, because if we don’t entertain them by talking to them, we will not have a good relationship with them which could affect our work in the village. But by knowing each other, that relationship has contributed immensely for people to come and take the medicine. For example, by knowing each other, any of them who pass nearby, and I asked him or her to come and drink the medicine, he will come and take it. – Fieldworker.

As demonstrated in the quote, fieldworkers knew the nuances of their relationships with the individuals in the communities were key to a successful trial. *‘Greeting’* and *‘entertaining elders’* in the ways that were appropriate to this specific context and to these specific villages were some of the learned, tacit skills fieldworkers developed as a way to do their job well. The success of MASSIV – and to some extent the security of the fieldworkers’ jobs – was dependent on trial indicators such as enrollment and coverage/compliance numbers. In this way, many fieldworkers felt the trial villages held considerable power. To paraphrase one fieldworker from an informal discussion: *The fieldworkers do not hold power; the communities hold all the power. The only power a fieldworker has is his attitude and using that to get the communities to trust him so that they take the medication*.

In addition, it became apparent through our ethnographic research that an individual fieldworker’s ability to influence people to take the medication varied across trial villages and was affected by the social dynamics of the given village. In certain villages with strong leadership from the Alkalo and the compound heads, the fieldworkers could rely on internal social dynamics, such as social and familial pressures, to help increase and maintain MDA involvement. However, in villages where the direction of the Alkalo and compound heads was not as impactful, the interpersonal relationships the fieldworkers established with the community members were all the more important in encouraging individuals to take the medication. Had the fieldworkers not built up these relationships – through showing respect and establishing trust – people in these villages would be less inclined to participate in the MDA.

## Discussion

This study has demonstrated the complexities of the body work conducted by fieldworkers in the context of an MDA clinical trial for malaria elimination in the Gambia. As is often the case, fieldworkers did far more than administering, recording and monitoring the trial medication on the bodies of the individuals in the trial villages: they manoeuvred, shaped, and maintained nuanced, reciprocal relationships among themselves, the trial participants, the MRCG and the trial villages. Working within the micro-political relations of body work (Twigg et al., 2011), they showed respect and established trust through their day-to-day labour and activities on the bodies of participants in a manner that can be defined as ‘reciprocity work’.

Much of the literature regarding reciprocity as it relates to health research and clinical trials focuses on the informed consent process and the role of the (foreign) individual researcher and the (local) individual participant. It emphasises how creating a reciprocal dialogue makes the relationship more equal and can increase autonomy in the informed consent process and aid in creating more ethical research (Bourdieu, 1986; Maiter et al., 2008). Further, reciprocity has been established as essential to the success of clinical trials, particularly within low-resource contexts in the Global South (Dada et al., 2019; Kerasidou, 2017). Especially in more communal cultural settings, reciprocity is a way to ensure there is not overdependence on one person or group. It is a way to share in good fortune, grief and material goods; there is an expectation that if one shares with you, you will share with them at a later time (Ujewe, 2018). Within the context of clinical trial research in the Gambia, where relationships of reciprocity are integral to maintaining individual relationships as well as overall community social cohesion (Fehr, Muela, et al., 2021; Fehr, Nieto-Sanchez, et al., 2021), studies have demonstrated the existence of relationships of ‘exchange’, where trial participation was given for medical care (Geissler et al., 2008). In these instances, the reciprocity work conducted by the fieldworkers was a critical element of the trials’ success.

This study elaborates on previous research by demonstrating that reciprocal relationships, and the reciprocity work conducted to maintain them, are formed within a greater historical context, and are imbued in – existing and trial induced – power dynamics amongst all actors within the greater trial community, including the MRCG, the fieldworkers and the individuals in the trial villages. These power dynamics are not a characteristic of a particular person or group, but are produced within the relationship itself, as well as the ‘material, social, and normative’ context (Broer et al., 2012; Foucault, 1978; Tourney, 1983) Within research relationships, in particular, power is created – and reinforced – by making it a part of the ‘norm’ (Heroux, 2001; Kerasidou, 2017)

In this study, relationships of power and of reciprocity operated in several directions based upon the actors involved. The most obvious holder of power is the MRCG, a large institution with international ties and access to funding. Within this context, where basic healthcare is difficult to access, the MRCG is able to contribute to the reciprocal relationship by providing ancillary care through trials and treatment through their clinics. This has been a reason for some individuals in the trial villages to participate in the MRCG trials or do ‘*whatever they ask*’. Between the MRCG and the fieldworkers, the MRCG is a provider of highly sought-after employment and a steady income. But, as shown by Kingori and Gerrets (2019), fieldworkers also hold power in their ‘localness’. Institutions, including the MRCG, rely upon fieldworks to conduct their reciprocity work in such a way that also allows them to operate as cultural or linguist ‘brokers’ and ‘intermediaries’ between the institution and the trial villages (Kingori & Gerrets, 2019).

There was, however, nuance in the perspectives of who held power in these relationships and how or in what point of time that power was more prevalent. Several of the MASSIV fieldworkers, as fieldworkers in other studies (Gikonyo et al., 2008; Kingori, 2013) recognised how they may hold positions of power the dynamics between themselves and the individuals in the trial villages, furthered by knowing that they had greater access to wealth and material goods. This was reflected by their need to show that they are ‘on *their level’* and not acting ‘too *big’* – small aspects of conducting reciprocity work. Further, through their employment with MRCG, fieldworkers may also have held power in the villages as the individual suppliers of healthcare. Though not noticed in this study, this particular power differential creates the potential for exploitation: in order to reach the coverage ‘quota’ required by their employer, fieldworkers could potentially take advantage of the villagers’ need for healthcare. On the other hand, many fieldworkers believed that the villagers wield considerable power. If villagers showed no or limited inclination to participate and take the MDA medication, the fieldworkers would not be able to do their jobs successfully and may lose future employment, and the MRCG may not be able to conduct the clinical trials required to maintain their standing within the Global Health community.

The reality in the field is complex and relationships of reciprocity – and the actions of reciprocity work – strongly affect individual and communities’ autonomy to participate in clinical trials. By providing paracetamol with their own money, for example, fieldworkers increased trust in the communities and also increased participation in the MDA. In addition to recognising this as a necessity, as well as a justice issue, the fieldworkers knew that if they were unable to provide medication to alleviate the side effects, people would not continue taking the medications in this trial and would be less likely to participate in future trials. As a single event representing the historical context and power/resource imbalances, reciprocity work in the sense of providing additional care may have increased participation, but may have also undermined autonomy by making those who received medical care feel they ‘must’ participate to continue to have access. In other trials conducted by MRCG in the Gambia, there was an expectation that by providing, for example, blood samples, study participants and their families would be given medical care (Geissler et al., 2008). In this context, it is possible that a lack of healthcare infrastructure has led to an expectation of reciprocity in the form of immediate and tangible trial benefits (Kelly, 2011), which could lead to structural coercion (Fisher & Fisher, 2020; Nyirenda et al., 2020).

Like all research institutions following international research ethics guidelines, the MRCG operates its trials with the policy of individual informed consent free of undue influence. The disconnect between ethics guidelines and what actually takes place ‘in the field’ has been documented in many settings (Gikonyo et al., 2008; Kamuya et al., 2015; Kingori, 2013, 2015). However, it is the fieldworkers who must navigate the tension between what they are expected to do per protocol and what is more ‘culturally appropriate’ in order to meet trial demands (such as securing adequate coverage). In this way, fieldworkers often have to ‘make ethics’ (Kingori, 2013) in a way that contradicts those formalised in Global Health research. For example, as part of their reciprocity work, some fieldworkers were involved in continuous dialogue with community members and gave them a space to ask questions regarding the trial and its medications. But by strengthening the interpersonal relationships, these acts of reciprocity work may also undermine autonomy in that the individuals in the trial village may feel they ‘owe’ the fieldworker their participation (Vincent et al., 2022). Additionally, in the event of the young woman who wanted to wait for her mother’s approval, the fieldworkers were following ethical protocol: she was over the age of 18 and did not need parental consent to participate in the trial. In theory, she had the agency to make her own decision regarding participation. In pushing her enrolment, the fieldworkers lost a potential trial participant, and the woman lost the potential benefits of trial participation. Had the fieldworkers done the more culturally appropriate thing – and showed respect by allowing her to consult with her mother for her approval first – the woman may have enrolled in the trial. On the other hand, however, they may have also undermined the trial protocol and general research ethics.

## Conclusion

By beginning with the concept of body work as it relates to the roles of fieldworkers in an MDA clinical trial, this study has demonstrated that fieldworkers partake in a multitude of day-to-day interactions, job responsibilities and balancing of complicated relationships in what can be called reciprocity work. There are three main components of this relationship. First, the fieldworkers are obligated to the trial and to their employer to achieve high MDA coverage. Second, fieldworkers need to maintain personal relationships with the individuals in the trial villages. In doing so, they must act in accordance with the social norms and expectations that this reciprocity entails. For their work to be fulfilled, they rely upon the individuals to comply with their requests of participation. In return, they may supply individuals with the vital healthcare and medications they need and cannot otherwise easily access. Third, fieldworkers act as representatives of the MRCG and maintain the long-standing reciprocal relationship between the institution and Gambian villages. Reciprocity work has implications for both trial coverage and individual autonomy in the informed consent process, as it can both increase and decrease autonomy in participation. The nuanced roles and myriad skills of the fieldworkers shape not only the success of the specific trial itself but also research ethics in general. In addition to studying the ethics of neocolonial relations in Global Health research, studying the reciprocity work of fieldworkers is also important for understanding how research ethics are shaped on the ground.
